# Functional coordination between leaf traits and biomass allocation and growth of four herbaceous species in a newly established reservoir riparian ecosystem in China

**DOI:** 10.1002/ece3.4494

**Published:** 2018-11-08

**Authors:** Gaoming Xiong, Aiying Zhang, Dayong Fan, Jielin Ge, Dan Yang, Zongqiang Xie, Wangfeng Zhang

**Affiliations:** ^1^ The Key Laboratory of Oasis Eco‐agriculture Xinjiang Production and Construction Corps Shihezi University Shihezi China; ^2^ State Key Laboratory of Vegetation and Environmental Change Institute of Botany Chinese Academy of Sciences Xiangshan, Beijing China

**Keywords:** growth analysis, relative growth rate, riparian ecosystem, Three Gorges Reservoir Area

## Abstract

The flood‐dry‐flood cycle in the reservoir riparian zone (RRZ) of the Three Gorges Dam has dramatically altered the riparian ecosystem structure and composition. Previous field studies have shown that leaf traits varied greatly and were restricted to the lower‐investment and faster‐return end of the global leaf spectrum, which are typical characteristics of fast‐growing species. However, it is unclear as to the mechanism underpinning the growth potential of these species and how it will respond to soil nutrient availability and temperature. Here, we linked the plant functional traits of four representative dominant C_4_ herbaceous species (*Setaria viridis*,* Echinochloa crusgalli*,* Cynodon dactylon* and *Hemarthria altissima*) to their relative growth rates (RGR) under ambient and elevated temperatures, with different nitrogen and phosphorus levels, to explore the potential mechanism of species growth in the newly established reservoir riparian ecosystem in the Three Gorges Reservoir Area, China. We grew seedlings of these species in four open‐top chambers, with three levels of nutrient supplies under two temperature gradients (ambient temperature and an elevated temperature of 4°C). We found that the responses of the RGR and plant traits to soil N and P supply levels and temperature varied considerably among studied species. *E. crusgalli* displayed the lowest RGR associated with relatively low specific leaf area (SLA), leaf nitrogen content (LN), stem mass ratio (SMR), and high leaf mass ratio (LMR) and was less affected by soil N and P supply levels and temperature. *C. dactylon* and *H. altissima* showed the highest RGR compared to the other two species grown at the substrate of N = 0.4 mg/g, P = 0.2 mg/g at ambient air temperature, associated with a relatively high SMR, low LMR and low plant carbon content (PCC). However, the RGR advantage of the two species was diminished at elevated temperatures, while *S. viridis* showed the highest RGR compared to the other species. Across all datasets, the RGR had no association with the leaf area ratio (LAR) and SLA. The RGR also showed no significant relationships with the LN and leaf phosphorus content (LP). On the other hand, the RGR was captured adequately by the SMR, which can therefore be considered as a powerful functional marker of species’ functioning in this newly established reservoir riparian ecosystem. Our study provides some insight into the underlying mechanisms of species growth in reservoir riparian ecosystems.

## 1 INTRODUCTION

The Three Gorges Dam (TGD) is the largest dam in China (Wu et al., 2004). Water levels in the reservoir vary from 175 m above sea level (a.s.l.) in winter to 145 m a.s.l. in summer, with a short exposure time of an average of 130 days per year at 158 m a.s.l. (Fan, Xiong, Zhang, Liu, & Xie, 2015). Since its operation at full capacity in 2007, the TGD has dramatically transformed the original plant communities to newly established reservoir ecosystems. The original plant communities have been gradually replaced by those dominated by perennial and annual herbaceous species (Fan et al., 2015; Wang et al., 2012; Ye, Zhang, Deng, & Zhang, 2013). Therefore, the detailed knowledge of the eco‐physiological processes underlying their dominance is critical to understanding the community assembly mechanism of this riparian ecosystem, as well as improving restoration practices (Fraaije, Braak, Verduyn, Verhoeven, & Soons, 2015; Garssen, Baattrup‐Pedersen, Voesenek, Verhoeven, & Soons, 2015; Shafroth, Perry, Rose, & Braatne, 2016).

Jie, Fan, Xie, Zhang, and Xiong (2012) have measured the leaf traits of 42 species in the riparian zone of the Three Gorge Reservoir Area (TGRA) and found that these traits were restricted to the lower‐investment and faster‐return end of the global leaf spectrum, which are typical characteristics of fast‐growing species (Wright et al., 2004). Fast‐growing species would have advantages in terms of competition for space and/or limiting nutrients, as would those species with rapid completion of their life cycle during the time they are exposed in riparian ecosystems, whereas flooding periods are survived by dormant life stages (Junk, 1993; Menges & Waller, 1983; Schöngart et al., 2007), which has been observed in some riparian ecosystems worldwide (Junk, 1993; Schöngart et al., 2007). As an example, the dominant species *Echinochloa polystachya* in the Amazon floodplain is the most productive and fastest growing emergent macrophytes in the world (Junk, 1993). However, it is worth noting that the means by which hydrological characteristics interact with and affect the riparian zone are complex and often unique to individual rivers (New & Xie, 2008). For instance, the riparian plants in the southwestern desert in North America adopted a “stress tolerant” strategy characterized by slow growth, instead of a “ruderal” strategy characterized by fast growth (Smith, Devitt, Sala, & Cleverly, 1998).

In addition to the shortened “growth window,” the herbaceous species also face the challenge of altered soil nutrient availability and elevated air temperature in the newly established reservoir riparian ecosystems in the TGRA. With regard to soil nutrient availability, it was observed in the field that along the altitude gradient, the lowland site (145–155 a.s.l.) appeared to be associated with higher phosphorus and lower nitrogen contents compared with the highland site (156–175 a.s.l.) (Ye et al., 2013). Such a soil nutrient gradient largely impacts the species composition and occurrence at different sites (Ye et al., 2013). In terms of temperature, the previous study has shown that since the operation of the TGD at full capacity in 2007, the average annual temperature in the TGRA has increased by 0.4–1.2°C (Zhong, Gao, & Yang, 2010). Furthermore, herbaceous species in the TGRA riparian ecosystems experience high‐temperature stress in the summer, mainly due to the absence of a “shade effect” by the diminishment of shrubs and trees. In fact, treeless riparian areas could increase maximum daily air temperatures by 3.2–3.4°C, compared to the adjacent riparian forests (Meleason & Quinn, 2004).

The Relative Growth Rate (RGR) is the most commonly used metric for quantifying and comparing the intrinsic growth potential among species (Liu et al., 2016; Oguchi, Ozaki, Hanada, & Hikosaka, 2016; Tomlinson et al., 2014). The RGR is regarded as one of the most important components of plant fitness (Hunt, 1982). Prior studies have indicated that the RGR varies considerably among plant species and is associated with a wide variety of parameters related to physiology, morphology, biomass partitioning and chemical composition (Grime, 1979; Lambers & Poorter, 1992; Wright et al., 2004). Linkages between plant functional traits and the RGR could provide new insight into the possible mechanism(s) of species’ adaptability in riparian ecosystems (Merritt, Scott, Poff, Auble, & Lytle, 2010). For example, depending on the daily quantum input (DQI), there was either a positive (Poorter & Remkes, 1990; Poorter & van der Werf, 1998) or no correlation (Li, Suzuki, & Hara, 1998; Oguchi et al., 2016) between the specific leaf area (SLA) and the RGR, demonstrating the changing role of leaf light‐interception capacity on the RGR under variable light conditions (Shipley, 2006). Since shrubs and trees decreased after the closure of dam, herbaceous species were exposed to full sun during the growing season. Therefore, we hypothesized that SLA was not coupled with the RGR in the newly established reservoir riparian ecosystem.

Although there is a large body of the literature on the physiological consequences experienced by riparian species due to tremendous water‐level fluctuations in the TGRA (Lei, Zeng, Xu, & Zhang, 2017; Li et al., 2011, 2015; Lu, Li, Jiang, Huang, & Bao, 2010), the responses of RGR to environmental variables, and the functional coordination between plant traits and the RGR in the newly established ecosystem, have not been fully explored. In the present study, we examined the RGR responses to elevated temperature and soil nutrient status and the link between plant functional traits and the RGR in four C_4_ herbaceous species under controlled conditions. The following species were used in this study: *Setaria viridis*,* Echinochloa crusgalli*,* Cynodon dactylon,* and *Hemarthria altissima* (Table 1). These species belong to the family Poaceae and have a C_4_ photosynthetic pathway (Li et al., 2015; Zhang, Fan, Li, Xiong, & Xie, 2016). *S. viridis* and *E. crusgalli* are annuals, while *C. dactylon* and *H. altissima* are perennials. These four herbaceous species co‐occurred and were predominant at different sites in the riparian zone formed by the TGRA in China (Wang et al., 2012; Zhang et al., 2016). Specifically, we aimed to: (1) determine how the RGR responds to soil nutrient status and temperature and whether it be applied to explain the species distribution pattern in the riparian ecosystem in TGRA, and (2) determine which plant functional traits explained the most variation in the RGR.

**Table 1:** Species used in this experiment

Species	Life form	Genus
*Echinochloa crusgalli* (L.) Beauv.	Annual	*Echinochloa*
*Setaria viridis* (L.) Beauv.	Annual	*Setaria*
*Cynodon dactylon* (L.) Persoon	Perennial	*Cynodon*
*Hemarthria altissima* (Poir.) Stapf et C. E. Hubb.	Perennial	*Hemarthria*

## 2 MATERIALS AND METHODS

### 2.1 Plant materials and growth conditions

We collected seeds from the four species during the autumn of 2013. The seeds were sown on wet paper placed over petri dishes and placed in growth chambers with 25°C/20°C day/night temperatures in May 2014. After germination, 20 seedlings were planted in 12 cm × 9 cm (height × diameter) pots filled with river sand, prewashed with deionized water. Sand was adopted as the substrate in the present study because the cation exchange capacity would be reduced by sand. Consequently, the soil total nitrogen (N) and total phosphorus (P) fed to plants would be similar to those in the soil solution in the pots (Garrish, Cernusak, Winter, & Turner, 2010).

We set up three N:P supply levels to mimic the N and P concentrations at sites along an altitudinal gradient in the TGD riparian ecosystem [N = 1 mg/g, P = 0.1 mg/g for the upland (174 m a.s.l.) treatment, with an N:P ratio = 10; N = 0.5 mg/g, P = 0.1 mg/g for the middle‐land (165 m a.s.l.) treatment, with an N:P ratio = 5; and N = 0.4 mg/g, P = 0.2 mg/g for the lowland (156 m a.s.l.) treatment, with an N:P ratio = 2] (our unpublished data). The N was supplied to the nutrient solutions as NH_4_NO_3_, and P was supplied as KH_2_PO_4_.

We applied Hoagland solution to each pot. The pH of each solution was 7.0 ± 0.1. Nutrient solutions were prepared with deionized water. Each pot received 0.3 L of the nutrient solution three times per week. The moisture in the sand was monitored every day, and sometimes, we added a small amount of water to avoid water stress.

We used four open‐top chambers (OTC) to explore the impacts of elevated temperature on plant growth. Each OTC covered a basal area of π × (1.5 m)^2^ and was 2.0 m high. The chambers were made of 8‐mm thick UV‐resistant polycarbonate, which reduced photosynthetic‐active radiation by 20% (daily quantum input ranged from 26–37 mol·m^−2^·day^−1^, measured by a LI190s quantum sensor). The air temperature in each OTC was controlled by an air‐conditioner. Two OTCs were set to increase the temperature in the chamber by 4°C at 10 cm above ground (+4°C); another two were set to maintain the ambient temperature in the chamber (ambient). Temperature records from each minute during the experimental period showed that daily average temperature in the +4°C OTCs was higher than ambient temperature by 3.84°C, while the temperature in the ambient OTCs was not significantly different from that of the surroundings. During the experiment period, the ambient average air temperatures at noon and midnight were 29.10°C and 19.05°C, respectively. A vertical temperature gradient (from 10 cm up to 110 cm above the ground) was measured in each OTC, and there was approximately a 0.1°C variation for both ambient and +4°C treatments.

We placed a total of 240 pots (3 N:P supply levels × 2 temperatures × 4 species × 10 pots) in the four OTCs. In each OTC, pots were randomly placed, and pot positions were changed periodically. Plants in the pots were allowed to adapt to the growth conditions for 1 month. After the initial plant, dry mass (InitBiomass) was measured on July 1st, 2014, excess plants were removed to maintain one healthy individual per pot. Although the pot was not large, plants could develop root systems through the hole at the bottom of the pot. Thus, we considered that pot size did not limit plant growth. We also monitored the ontogeny of the four species at two growth temperatures, and they did not show buds during the experimental period. Additionally, there was no significant relationship between the RGR and mean plant dry mass at the end of the experimental period (FinalBiomass) (*r* ^2^ = 0.225, *F* = 3.173, *p* = 0.120, at ambient temperature; *r* ^2^ = 0.151, *F* = 1.630, *p* = 0.219, at +4°C). There was also no significant relationship between the RGR and mean plant dry mass at the beginning of the experiment (*r* ^2^ = 0.272, *F* = 2.617, *p* = 0.150, at ambient temperature; *r* ^2^ = 0.299, *F* = 4.274, *p* = 0.066, at +4°C). Therefore, we assumed that both the ontogeny shift and plant size had limited impacts on the following growth analysis.

### 2.2 Relative growth rate, biomass allocation, and leaf morphological traits

On July 1st, 2014, we estimated the initial plant dry mass by harvesting 14–16 individuals from each treatment. At the end of the experiment (from July 29th to August 1st), leaves, stems, and roots of 6–8 individuals were harvested separately and dried to a constant weight at 70°C for 72 hr. The RGR was calculated as [log_e_(*M2*) − log_e_(*M1*)]/*t*, where log_e_(*M2*) and log_e_(*M1*) are the natural logarithms of the mean plant dry mass at the end (Final Biomass) and beginning (Init Biomass) of the experiment, respectively, and *t* is the duration of the experiment.

The leaf mass ratio (LMR) was calculated as leaf mass/total biomass, the stem mass ratio (SMR) was calculated as stem mass/total biomass, and the root mass ratio (RMR) was calculated as root mass/total biomass. The leaf area ratio (LAR) was calculated as LAR = SLA × LMR, while SLA is the specific leaf area.

Mature organs (leaves, stems, and roots) from 3 to 5 individuals were collected separately for further measurements of C, N, and P concentrations.

### 2.3 Plant C, N, and P concentration measurements

Each sample was ground to a fine powder after drying, using a ball mill. The C and N concentrations were analyzed by the Dumas combustion method, using an elemental analyzer (Elementar vario EL III, Elmentar, Hanau, Germany). We determined the P concentration by an inductively coupled plasma optical emission spectrometer (Thermal 6300, Thermo Scientific, West Palm Beach, FL, USA) after acid digestion. The plant's carbon concentration was PCC = RMR × RC + SMR × SC + LMR × LC. The definitions for the aforementioned abbreviations can be found in Table 2.

**Table 2:** List of measured and calculated parameters in the text

Abbreviation	Definition	Unit	Method
InitBiomass	The first harvest biomass	g	Measured
FinalBiomass	The second harvest biomass	g	Measured
SLA	Specific leaf area: leaf area per unit leaf mass	m^2^/g	Measured
LC, SC, RC	Leaf, stem and root carbon concentration, respectively	mg C/g	Measured
LN, SN, RN	Leaf, stem and root nitrogen concentration, respectively	mg N/g	Measured
LP, SP, RP	Leaf, stem and root phosphorus concentration, respectively	mg P/g	Measured
LMR	Leaf mass ratio: ratio of leaf mass to plant dry mass		Measured
SMR	Stem mass ratio: ratio of stem mass to plant dry mass		Measured
RMR	Root mass ratio: ratio of root mass to plant dry mass		Measured
RGR	Relative growth rate	g·g^−1^·day^−1^	Calculated
LAR	Leaf area ratio	m^2^/g	Calculated
PCC	Plant carbon concentration	mg C/g	Calculated

### 2.4 Data analysis

Data were tested for normality and homogeneity of variance before analysis and were log_10_‐transformed when necessary (for SLA, SMR). Differences among treatments (3 N:P supply levels × 4 species × 2 temperatures) were tested by a three‐way analysis of variance (ANOVA) and an LSD post hoc test. The differences between life forms, and their interactions with soil and temperature, were also tested by an ANOVA.

For the RGR calculation and comparison, we simulated 1000 posterior estimates of M2 and M1 from the fitted models for each species with each stratum (i.e., temperature and N:P supply levels). We then calculated the product of each pair of simulated probabilities to yield a posterior distribution for the RGR. Significant differences in the RGR among temperature and N:P supply levels were tested by bootstrapping a 95% confidence interval for the difference between strata means and determining if the interval overlapped zero (McCarthy, 2007). We then performed the three‐factor analysis of variance procedure to determine the interaction effects of species, soil, and temperature on RGR.

Since the LAR, SLA, LMR, PCC, RMR, PNC, LN, and LP are regarded as directly associated with the RGR (Hunt, 1982; Oguchi et al., 2016), we also conducted a Pearson's correlation analysis between these parameters and the RGR. We conducted all statistical analyses in R version 3.4.0 (R Development Core Team 2016) or SPSS 17.0 (SPSS, Chicago, IL, USA).

## 3 RESULTS

### 3.1 The leaf morphological traits and biomass allocation

**Figure 1:**
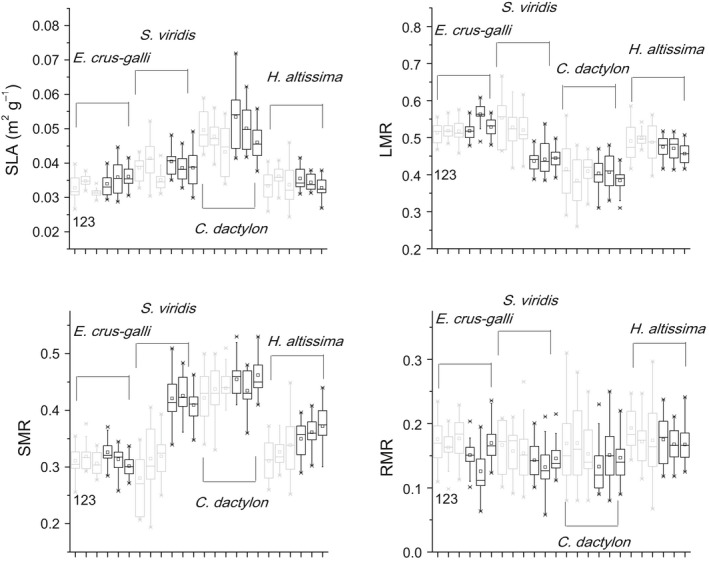
The boxplots of specific leaf area (SLA), leaf mass ratio (LMR), stem mass ratio (SMR), and root mass ratio (RMR) in four C_4_ grass species at ambient and +4°C temperatures. The midpoint of a boxplot is the median. The 25% and 75% quartiles define the low and high hinges, respectively. Lines are drawn from each hinge to 1.5 times the spread or to the most extreme value of the spread, whichever is smaller. Gray boxes and black boxes represent values of plants growing at ambient and +4°C temperatures, respectively. 1, 2, and 3 in each subfigure represent plants growing with supply of N = 0.4 mg/g, P = 0.2 mg/g (1); N = 0.5 mg/g, P = 0.1 mg/g (2); and N = 1 mg/g, P = 0.1 mg/g (3), respectively

The leaf morphological traits and biomass allocation (SLA, LMR, SMR, and RMR) were species‐dependent. Among the four species, *C. dactylon* had the highest SLA (0.048 m^2^/g), the highest SMR (0.44), and the lowest LMR (0.40); *E. crusgalli* displayed the highest LMR (0.53) and the lowest SMR (0.31); and *H. altissima* exhibited the highest RMR (0.17) (Figure 1). Elevated temperature increased the SLA and SMR but decreased the LMR and RMR significantly. Soil with N = 1 mg/g, P = 0.1 mg/g had significantly lower SLA values than the other two soil treatments. The Sp (species) × Soil interaction significantly affected the SLA, SMR, and RMR. The T (temperature) × Soil interaction significantly influenced the SLA, LMR, SMR, and RMR. The T × Soil interaction significantly affected the LMR, SMR, and RMR (Table 3). Life form significantly affected the morphological traits, with perennials having a higher SLA (*F* = 46.94, *p* < 0.001), lower LMR (*F* = 148.53, *p* < 0.001), higher SMR (*F* = 82.192, *p* < 0.001) and higher RMR (*F* = 7.89, *p* = 0.005) than annuals. There were no significant interactions between life forms and other factors (soil or temperature) for the morphological parameters, except for a significant interaction effect of Life form × Temperature on the SMR (*F* = 9.30, *p* = 0.002).

**Table 3:** The leaf/stem/root carbon concentrations (LC, SC, RC), leaf/stem/root nitrogen concentrations (LN, SN, RN), and leaf/stem/root phosphorus concentrations (LP, SP, RP) in four C_4_ grass species at Ambient and +4°C temperatures

Species		Ambient			+4°C		
		N:P = 2	N:P = 5	N:P = 10	N:P = 2	N:P = 5	N:P = 10
		Carbon concentration					
*E. crusgalli*	Leaf	419.9 ± 6.1	424.0 ± 5.6	419.8 ± 5.5	422.5 ± 5.8	425.6 ± 8.7	423.7 ± 5.7
	Stem	381.5 ± 6.9	373.4 ± 6.5	374.8 ± 6.4	375.9 ± 4.7	374.5 ± 6.3	373.5 ± 6.4
	Root	394.1 ± 4.7	387.5 ± 4.3	390.7 ± 4.7	396.7 ± 4.5	388.3 ± 4.8	392.8 ± 4.3
*S. viridis*	Leaf	389.3 ± 5.6	396.5 ± 5.7	399.3 ± 5.2	401.1 ± 6.2	402.5 ± 5.3	404.1 ± 5.7
	Stem	346.4 ± 5.7	355.2 ± 5.7	355.9 ± 6.1	364.4 ± 5.8	368.9 ± 6.2	365.8 ± 5.7
	Root	393.8 ± 4.8	402.0 ± 3.9	394.4 ± 3.7	404.0 ± 4.2	397.0 ± 4.8	405.0 ± 5.9
*C. dactylon*	Leaf	419.8 ± 5.7	416.9 ± 5.8	413.8 ± 6.7	418.2 ± 6.1	415.6 ± 5.8	414.2 ± 5.6
	Stem	390.6 ± 6.8	379.1 ± 6.9	382.9 ± 6.6	381.8 ± 9.0	381.1 ± 7.3	382.5 ± 6.3
	Root	389.9 ± 4.6	384.5 ± 4.1	395.5 ± 4.9	369.1 ± 3.8	378.8 ± 7.7	396.4 ± 3.8
*H. altissima*	Leaf	410.7 ± 5.2	412.9 ± 5.6	411.6 ± 5.8	411.5 ± 5.3	413.8 ± 5.6	414.1 ± 5.9
	Stem	387.2 ± 6.8	386.1 ± 7.0	382.5 ± 7.3	389.4 ± 7.4	391.4 ± 6.8	392.1 ± 6.6
	Root	395.3 ± 4.1	390.8 ± 4.7	381.6 ± 4.2	377.8 ± 6.5	394.6 ± 4.2	381.6 ± 4.5
		Nitrogen concentration					
*E. crusgalli*	Leaf	28.7 ± 2.0	30.6 ± 2.1	31.2 ± 2.1	26.2 ± 1.9	28.7 ± 2.2	31.9 ± 2.2
	Stem	21.0 ± 1.6	24.9 ± 2.0	27.1 ± 2.0	18.4 ± 3.0	18.8 ± 1.3	24.2 ± 2.1
	Root	12.5 ± 1.0	16.3 ± 1.4	17.2 ± 1.4	9.7 ± 1.0	12.2 ± 1.3	15.1 ± 1.5
*S. viridis*	Leaf	36.8 ± 2.5	35.8 ± 3.0	35.5 ± 4.6	30.9 ± 2.8	29.2 ± 2.8	33.9 ± 2.9
	Stem	34.8 ± 2.8	34.1 ± 2.8	34.7 ± 2.7	24.5 ± 2.1	22.2 ± 1.8	27.8 ± 2.4
	Root	22.8 ± 1.9	22.9 ± 2.2	23.1 ± 2.0	16.4 ± 1.8	15.1 ± 1.2	18.6 ± 1.6
*C. dactylon*	Leaf	37.5 ± 2.5	40.9 ± 3.0	39.2 ± 4.6	41.2 ± 2.8	40.6 ± 2.8	38.0 ± 2.9
	Stem	22.0 ± 1.7	28.8 ± 2.4	28.5 ± 2.5	25.5 ± 1.2	26.4 ± 2.1	25.6 ± 2.1
	Root	16.0 ± 1.4	17.9 ± 1.4	19.0 ± 1.5	14.7 ± 1.2	17.0 ± 1.3	16.6 ± 1.5
*H. altissima*	Leaf	29.7 ± 1.9	30.9 ± 2.2	29.7 ± 2.1	27.4 ± 2.0	26.6 ± 1.8	26.2 ± 1.8
	Stem	23.1 ± 1.6	26.0 ± 2.2	23.7 ± 1.9	20.6 ± 1.7	20.1 ± 1.7	19.9 ± 1.6
	Root	16.8 ± 1.6	19.5 ± 1.7	21.9 ± 1.8	17.5 ± 1.5	16.0 ± 1.4	19.4 ± 1.7
		Phosphorus concentration					
*E. crusgalli*	Leaf	3.21 ± 0.15	2.82 ± 0.17	2.65 ± 0.18	2.62 ± 0.16	2.56 ± 0.13	2.62 ± 0.14
	Stem	2.98 ± 0.24	2.57 ± 0.25	2.49 ± 0.21	2.42 ± 0.22	2.22 ± 0.16	2.50 ± 0.20
	Root	1.77 ± 0.11	1.65 ± 0.08	1.30 ± 0.07	1.31 ± 0.11	1.37 ± 0.08	1.33 ± 0.08
*S. viridis*	Leaf	2.63 ± 0.17	2.30 ± 0.18	2.29 ± 0.12	2.57 ± 0.15	2.22 ± 0.12	2.14 ± 0.08
	Stem	3.10 ± 0.23	2.16 ± 0.19	2.34 ± 0.18	2.21 ± 0.15	1.58 ± 0.15	1.48 ± 0.11
	Root	1.64 ± 0.11	1.59 ± 0.09	1.73 ± 0.10	1.47 ± 0.09	1.27 ± 0.06	1.19 ± 0.07
*C. dactylon*	Leaf	3.01 ± 0.20	2.66 ± 0.17	2.60 ± 0.16	3.05 ± 0.20	2.80 ± 0.17	2.41 ± 0.16
	Stem	2.63 ± 0.22	2.14 ± 0.22	2.15 ± 0.20	2.23 ± 0.15	1.87 ± 0.13	1.97 ± 0.14
	Root	1.64 ± 0.12	1.42 ± 0.07	1.23 ± 0.07	1.52 ± 0.09	1.51 ± 0.08	1.32 ± 0.07
*H. altissima*	Leaf	2.41 ± 0.13	2.42 ± 0.15	1.92 ± 0.09	2.36 ± 0.16	2.27 ± 0.11	2.17 ± 0.15
	Stem	2.20 ± 0.14	2.46 ± 0.19	1.86 ± 0.14	2.16 ± 0.19	2.15 ± 0.18	2.16 ± 0.15
	Root	1.43 ± 0.09	1.90 ± 0.14	1.54 ± 0.09	1.75 ± 0.09	1.62 ± 0.10	1.66 ± 0.09

### 3.2 The nutrient traits

The C, N, and P concentrations were species dependent. Among the four species, *S. viridis* displayed the lowest LC (398 mg/g) and SC (359 mg/g), but the highest RC (399 mg/g). Leaves of *C. dactylon* had higher LN (39.6 mg/g) compared with the other three species. *S. viridis* had the highest SN (29.6 mg/g) and RN (19.8 mg/g) among the species studied. *E. crusgalli* and *C. dactylon* had higher LP (2.74 mg/g and 2.76 mg/g) than the other two species. *E crusgalli* had the highest SP (2.53 mg/g), and *H. altissima* had the highest RP (1.65 mg/g) among the species studied. Elevated temperature significantly decreased the LN for the four species studied, with an average for the four species of 31.7 mg/g at +4°C, compared with 33.9 mg/g at ambient temperature. Temperature also decreased the SN (27.3 mg/g vs. 22.8 mg/g, at ambient temperature and +4°C, respectively) and RN (18.8 mg/g vs. 15.7 mg/g, at ambient temperature and +4°C, respectively). The different N and P supply levels in the soil significantly affected the LP, SN, SP, RN, and RP. Soil with N = 0.4 mg/g, P = 0.2 mg/g had higher LP (2.73 mg/g), higher SP (2.49 mg/g), higher RP (1.58 mg/g), lower SN (23.6 mg/g) and lower RN (15.8 mg/g) than soil with the other two N and P supply levels. Sp × T interaction significantly affected the SN, SP, RN, and RP. Sp × Soil interaction significantly affected the RC. There was a significant Sp × T × Soil interaction in terms of the RP concentration. Life form had no effect on the C, N, and P concentrations, except for perennials, which had a higher SC (*F* = 39.12, *p* < 0.001) and lower RC (*F* = 14.37, *p* < 0.001) than annuals. Life form × Temperature interaction significantly affected the RC (*F* = 4.93, *p* = 0.03) and RP (*F* = 12.12, *p* = 0.001). The concentrations of C, N, and P were highest in the leaves, lowest in the roots, and were in the middle for the stems (all *p* < 0.001) (Table 3 and 4).

**Table 4:** Effects (*F* value) of species, temperature and soil nutrient supply on specific leaf area (SLA), leaf mass ratio (LMR), stem mass ratio (SMR), root mass ratio (RMR), leaf/stem/root carbon concentrations (LC, SC, RC), leaf/stem/root nitrogen concentrations (LN, SN, RN), as well as leaf/stem/root phosphorus concentrations (LP, SP, RP) in four C_4_ grass species. *F* and *p*‐values (***p < 0.001; **p < 0.01; *p < 0.05) were determined by a two‐way analysis of variance (ANOVA)

Factors (*df*)	SLA	LMR	SMR	RMR
Sp (3)	273.39***	204.43***	272.46***	9.94***
T (1)	11.88***	44.22***	177.87***	26.01***
Soil (2)	21.74***	1.83^(ns)^	2.44^(ns)^	2.11^(ns)^
Sp × T(3)	2.04^(ns)^	36.72***	57.6***	0.73^(ns)^
Sp × Soil (6)	3.69**	1.94^(ns)^	3.42*	2.55*
T × Soil (2)	7.17**	3.49*	5.81*	2.62*
Sp × T × Soil (6)	2.02^(ns)^	1.80^(ns)^	1.24^(ns)^	0.52^(ns)^

Factors (*df*)	LC	LN	LP	SC	SN	SP
Sp (3)	17.4***	25.86***	17.43***	21.53***	16.48***	6.06*
T (1)	1.25^(ns)^	4.62*	2.26^(ns)^	1.99^(ns)^	27.39***	20.63***
Soil (2)	0.20^(ns)^	0.29^(ns)^	12.76***	0.05^(ns)^	3.53*	9.97**
Sp × T(3)	0.53^(ns)^	1.51^(ns)^	1.32^(ns)^	2.03^(ns)^	4.57**	4.38*
Sp × Soil (6)	0.36^(ns)^	0.67^(ns)^	0.64^(ns)^	0.43^(ns)^	1.28^(ns)^	2.37^(ns)^
T × Soil (2)	0.04^(ns)^	0.34^(ns)^	0.38^(ns)^	0.21^(ns)^	1.64^(ns)^	1.26^(ns)^
Sp × T × Soil (6)	0.07^(ns)^	0.4^(ns)^	0.98^(ns)^	0.21^(ns)^	0.39^(ns)^	0.64^(ns)^

Factors (*df*)	RC	RN	RP
Sp (3)	10.04***	16.81***	6.71**
T (1)	0.60^(ns)^	24.02***	11.49***
Soil (2)	0.47^(ns)^	7.81**	6.67**
Sp × T(3)	2.50^(ns)^	2.89*	6.83***
Sp × Soil (6)	3.46*	0.88^(ns)^	2.01^(ns)^
T × Soil (2)	2.06^(ns)^	0.59^(ns)^	0.95^(ns)^
Sp × T × Soil (6)	1.71^(ns)^	0.44^(ns)^	3.65*

### 3.3 Relative growth rate

Among the four species, *E. crusgalli* had the lowest RGR compared to the other three species, regardless of the temperature and nutrient treatments (Figure 3, Supporting information Table S1). At the ambient temperature, *C. dactylon* and *H. altissima* had higher RGRs than *S. viridis* and *E. crusgalli* under nutrient levels of N = 0.4 mg/g, P = 0.2 mg/g, and N = 0.5 mg/g, P = 0.1 mg/g. In contrast, under the nutrient level of N = 1 mg/g, P = 0.1 mg/g, the RGR of *S. viridis* was higher than that of *C. dactylon* and *H. altissima*. At the elevated temperature, *S. viridis* had a higher RGR than the other three species. There was no significant difference in the RGR between *C. dactylon* and *H. altissima*, except at the nutrient level of N = 0.5 mg/g, P = 0.1 mg/g at the elevated temperature.

Different species exhibited different responses to elevated temperature and nutrient treatments (Supporting information Table S2). Elevated temperature significantly increased the RGR of *S. viridis*, but there was no significant difference among soil nutrient levels. For *C. dactylon* and *H. altissima*, soil with N = 0.4 mg/g, P = 0.2 mg/g had a higher RGR than soil with N = 1 mg/g, P = 0.1 mg/g (β = 0.030, CI: 0.011, 0.050; β = 0.045, CI: 0.030, 0.061, respectively), at ambient temperature. The elevated temperature significantly increased the RGR of *C. dactylon* and *H. altissima* with the nutrient supply of N = 1 mg/g, P = 0.1 mg/g (β = −0.027, CI: −0.048, −0.006; β = −0.041, CI: −0.057, −0.023, respectively). However, elevated temperature had no significant impact on the RGR of the two species with the nutrient supply of N = 0.4 mg/g, P = 0.2 mg/g, and N = 0.5 mg/g, P = 0.1 mg/g. *E. crusgalli* had no significant responses in the RGR to the elevated temperature and nutrient treatments (Figure 3, Supporting information Table S2). Collectively, the Sp × T, Sp × Soil, T × Soil and Sp × T × Soil interaction significantly affected the RGR (*F* > 2909.30, *p* < 0.001 in all cases, Figure 3, Supporting information Table S1 and S2).

### 3.4 Relationships between traits and RGR

Across all dataset, the LAR and SLA were not significantly correlated with the RGR (*r* ^2^ = 0.01, *p* = 0.63 for SLA; *r* ^2^ = 0.08, *p* = 0.19 for LAR) (Figure 3). In terms of the relationships between biomass allocation traits and the RGR, the LMR showed a significantly negative relationship (*r* ^2^ = 0.27, *p* = 0.01), the SMR showed a positive one (*r* ^2^ = 0.30, *p* = 0.005), while the RMR showed no relationship with the RGR (*r* ^2^ = 0.08, *p* = 0.18) (Figure 4). There was a negative correlation between the PCC and RGR (*r* ^2^ = 0.11, *p* = 0.04), but no significant relationships were found between LN and the RGR (*r* ^2^ = 0.01, *p* = 0.68), or between LP and the RGR (*r* ^2^ = 0.13, *p* = 0.08) (Figure 5, Table 5). There was no difference in terms of correlation patterns between the ambient temperature treatment and elevated temperature treatment (*p* > 0.05 in all cases).

**Table 5:** Correlation coefficients (r) between seven traits associated with RGR

	RGR	LAR	SLA	LMR	RMR	SMR	PCC	LN	LP
RGR	1								
LAR	−0.102	1							
SLA	0.276	0.649**	1						
LMR	−0.516**	0.074	−0.706**	1					
RMR	−0.287	−0.512**	−0.494**	0.194	1				
SMR	0.556**	0.098	0.785***	−0.949***	−0.493**	1			
PCC	−0.379*	−0.338	−0.303	0.110	0.158	−0.148	1		
LN	0.089	0.625**	0.859***	−0.532**	−0.252	0.552**	−0.470*	1	
LP	−0.355	0.497**	0.418*	−0.042	−0.173	0.093	0.223	0.349	1

*p < 0.05; **p < 0.01;***p < 0.001.

## 4 DISCUSSION

### 4.1 Responses of the RGR and traits to soil N and P supply and temperature

**Figure 2:**
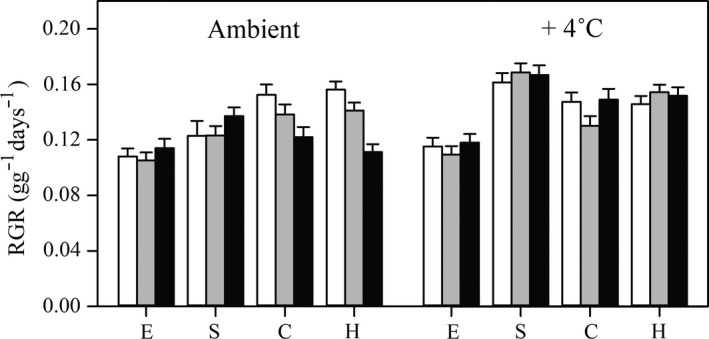
Relative growth rate (RGR) in four C_4_ grass species growing with different supplies of nutrients at ambient and +4°C temperatures. E, S, C, and H represent *Echinochloa crusgalli*,* Setaria viridis*,* Cynodon dactylon*, and *Hemarthria altissima*, respectively. Light gray, middle gray and dark gray bars represent the RGR of plants supplied with N = 0.4 mg/g, P = 0.2 mg/g (N2); N = 0.5 mg/g, P = 0.1 mg/g (N5); and N = 1 mg/g, P = 0.1 mg/g (N10), respectively. Bars represent ± standard error

In the present study, the responses of the RGR and traits to soil N and P supply levels and temperature varied considerably among the studied species (Tables 3 and 4; Figures 1 and 2; Supporting information Figure S1), indicating each species had its own solutions when subjected to altered nutrient availability and increased air temperature, even within the same plant functional group of the C_4_ photosynthetic pathway and belonging to the same family. For example, compared with the other three species, *E. crusgalli* had the lowest RGR (Figure 2) and was less affected by soil N and P supply levels and temperature; its low RGR was associated with a low SLA, LN, SMR and higher LMR (Figure 1, Table 3, Supporting information Figure S1). This is consistent with the observations that under either optimal or non‐optimal conditions, inherently fast‐growing species always have higher RGRs than slow‐growing species (Chapin, 1980; Shipley & Keddy, 1988).

The large variation of the responses of the RGR and traits under controlled environment would be helpful to understand local communities’ assembly in the newly established riparian ecosystem. For example, at the ambient air temperature, *C. dactylon* and *H. altissima* had the highest RGR compared to the other species grown in the substrate of N = 0.4 mg/g, P = 0.2 mg/g (lowland) (Figure 2). In parallel with the present experimental results, many field investigations have revealed that after the operation at full capacity in 2007, *C. dactylon* was the most common and dominant species in lowland areas in the riparian ecosystem of the TGRA (Fan et al., 2015; Li et al., 2015; Wang et al., 2012; Ye et al., 2013). This might suggest that a strong habitat filter effect favoring high RGR and its associated traits in lowland areas, consistent with the leaf traits’ measurement in the field (Jie et al., 2012).

Besides, along a flooding gradient in the riparian zone of the TGRA, field studies showed the dominance (coverage) of *C. dactylon* and *H. altissima* gradually decreased, while *S. viridis* increased from lowlands to highlands, after several years’ impoundment (Li et al., 2015; Liu, Wang, Wang, & Yang, 2012; Wang et al., 2012; Ye et al., 2013). In line with these findings, the results in the present study demonstrated that at the ambient air temperature, the RGR of *C. dactylon* and *H. altissima* gradually decreased from lowlands (N = 0.4 mg/g, P = 0.2 mg/g) to highlands (N = 1 mg/g, P = 0.1 mg/g), and there was a trend that *S. viridis* had a higher RGR in highlands (Figure 2). The accordance between the measured RGR under controlled conditions and the observation of species distribution and abundance highlights the RGR as one of the most important indicators of plant fitness (Grime & Hunt, 1975) in this newly established reservoir riparian ecosystem, the TGRA. In fact, a field observation also revealed that *C. dactylon* and *H. altissima* had higher RGRs in lowlands than highlands (Zhang, 2010), although it is hard to assess the ontogeny shift effect on the growth analysis in this study.

At the three nutrient supply levels and at the elevated temperature, *S. viridis* had, on average, a 29.7% higher RGR than at the ambient temperature, the largest increase for the species studied (Figure 2). This raises the possibility that *S. viridis* would tend to gradually dominate the riparian C_4_ communities in the TGRA under increased air temperature scenarios. However, this should be interpreted with caution. In addition to the traits investigated in the present study, other factors associated with temperature and nutrient including the availability of the soil seed bank (Lu et al., 2010), seed germination characteristics (Lin, Liu, Zeng, Pan, & Su, 2016), the priority effect of seed's arrival (Fraaije et al., 2015; Sarneel, Kardol, & Nilsson, 2016), the clonality of perennials (Li et al., 2015; Zhang, Fan, Xie, Xiong, & Li, 2010), the lateral spread ability after exposure to air (Wang et al., 2008; Xiong, Nilsson, Johansson, & Jansson, 2001), winter flooding resistance strategies etc., would also affect the position and abundance of species in the riparian zones. As such, the interactions between growth and other traits in the reservoir riparian ecosystem in the TGRA merit further study.

### 4.2 The functional coordination between the RGR and biomass partitioning, leaf nutrient content and leaf morphological traits

**Figure 3:**
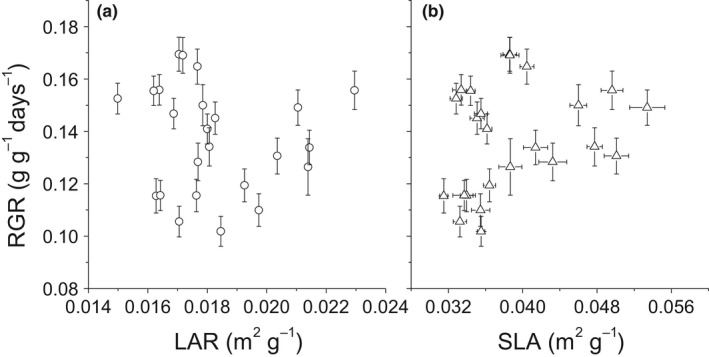
RGR as a function of: a) leaf area ratio (LAR), b) specific leaf area (SLA) of four herbaceous species with different nutrient supplies and at different growth temperatures. Bars represent ± standard error

The classic growth model in terms of carbon economy factors the RGR into two underlying components: LAR and NAR (the increase in biomass per unit leaf area), with RGR = NAR × LAR (Hunt, 1982). The LAR can subsequently be factored into the SLA (specific leaf area) and LMR, with LAR = SLA × LMR, indicating there may exist strong dependence of the RGR on SLA/LAR (Poorter & Remkes, 1990). However, in the present study, neither the SLA nor LAR had a significant correlation with the RGR (Figure 3), which is consistent with the findings of some earlier studies (Reich et al., 2003; Veneklaas & Poorter, 1998), but not with others (Poorter & Remkes, 1990; Poorter et al., 2005; Reich, Tjoelker, Walters, Vanderklein, & Buschena, 1998). In fact, in a meta‐analysis across 1,240 observations, Shipley (2006) found there was only a very weak correlation between the SLA and RGR. Furthermore, only at low irradiance (DQI < 15 mol·m^−2^·day^−1^) is the SLA the most important determinant of the RGR (Shipley, 2006). SLA/LAR was not linked to the RGR in the present study, indicating that competition for light resources was not a major factor limiting growth processes, because SLA/LAR is a key trait that reflects the capability of plants to intercept incident irradiance (Dwyer, Ghannoum, Nicotra, & Von Caemmerer, 2007; Sims, Gebauer, & Pearcy, 1994). This aligns with field observations that woody species gradually diminished after the dam was closed (Fan et al., 2015; Wang et al., 2012).

**Figure 4:**
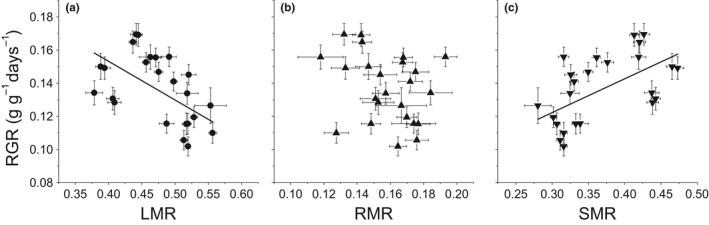
RGR as a function of: a) the leaf mass ratio (LMR), b) the root mass ratio (RMR), and c) the stem mass ratio (SMR) of four herbaceous species under different nutrient supplies and growth temperatures. The bold lines represent regression lines for statistically significant relationships. Bars represent ± standard error of the mean. Regression lines: *y *=* *−0.23*x* + 0.25 (a), *y *=* *0.20*x* + 0.06 (c)

The positive relationship between the SMR and RGR (Figure 4) among species was rarely reported (Villar, Veneklaas, Jordano, & Lambers, 1998). Villar et al. (1998) hypothesized that the positive relationship between the SMR and LAR among Poaceae species meant that a higher SMR was associated with a higher LAR, and consequently lead to a higher RGR because of the positive correlation between the LAR and RGR. Obviously, this was not the case in the present study (Figure 3; Table 5). Established plants can use avoidance strategies through the fast elongation of the stem in order to restore contact between leaves and air when submerged by flooding (Bailey‐Serres & Voesenek, 2008). However, this was not a plausible explanation in the newly established riparian ecosystem, since plants start their growth only after flooding events. Some studies have suggested that an ‘ideal successional dominant’ species starting on the exposed bottom of water reservoir is often a geophyte capable of intensive lateral spread (Keddy, 1999; Prach & Pyšek, 1999). In the current riparian setting, the field investigation has also found riparian individuals exhibited higher lateral expansion capacity and a higher SMR than non‐riparian individuals within species (Fan et al., 2015; Zhang et al., 2016). Possibly the capacity of intensive lateral spread and the associated high SMR is an advantage for dominant species to quickly forage for and colonize bare spaces after water recedes (de Kroons & Hutchings, 1995) and compete for limited nutrient resources (Grime, Hodgson, & Hunt, 1988) during the exposed period in order to support their high RGR in this newly established reservoir riparian ecosystem.

**Figure 5:**
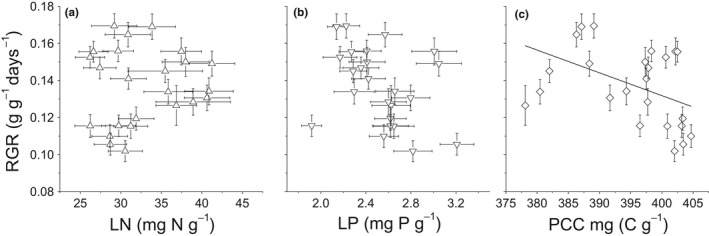
RGR as a function of: a) the leaf nitrogen concentration (LN), b) the leaf phosphorus concentration (LP), and c) the plant carbon concentration (PCC) of 4 herbaceous species under different nutrient supplies and growth temperatures. The bold line represents the regression line for statistically significant relationships. Bars represent ± standard error of the mean. Regression line: *y *=* *−0.0012*x* + 0.062 (c)

The lack of a relationship between the LN and LP and RGR (Figure 5, Table 5) might be related to the nitrogen and phosphorus supply levels; for example, under high‐phosphorus conditions, either luxury uptake or storage of P, or the flexibility in the investment in RNA to accomplish protein synthesis associated with growth, could obscure the relationship between the RGR and LP (Matzek & Vitousek, 2009). In the present study, the LN: LP ratios ranged from 8.9 to 15.8, with an average of 13.1. This finding indicated our studied species were more limited more by nitrogen than phosphorus (Güsewell & Koerselman, 2002). It is worth noting, however, that whether or not a plant's growth was N or P limited was species‐specific (Ågren, 2004). Therefore, the growth of each of the species studied may be limited by different elements, or by the same element but to different degrees. The former possibility can be partially excluded by the present study, as evidenced by the lack of a significant interaction with Sp × Soil and Sp × T × Soil in terms of LN, LP, SN, SP and RN (Table 4), but the latter possibility cannot be excluded and needs further investigation.

## 5 CONCLUSIONS

To the best of our knowledge, this is the first study to investigate the response of the RGR to soil nutrient availability and temperature in a newly established reservoir riparian ecosystem in China. We also have analyzed the functional coordination between leaves, biomass allocation traits and the growth of the four studied species. The RGR response to soil nutrient availability at ambient air temperatures was in good agreement with the studied species’ occurrence and distribution patterns observed in the reservoir riparian zone of the Three Gorges Dam. It was also found that the RGR was captured adequately by the SMR, which can therefore be considered as a powerful functional marker of species’ functioning in this newly established reservoir riparian ecosystem. Our study provided some insight into the mechanism(s) underpinning the adaptability of plants in the reservoir riparian ecosystem of the TGRA. However, closer attention needs to be paid to the interactions between growth and other biological processes in this newly established reservoir riparian ecosystem. Whether our conclusions could apply more generally in other riparian ecosystems warrants further investigation.

## AUTHORS CONTRIBUTIONS

GX, AZ, DF, WZ, ZX conceived the idea. GX, AZ, DF, DY and JG designed and performed the study. GX and AZ wrote the first draft and all the authors revised the manuscript. GX and AZ contributed equally.

## DATA ACCESSIBILITY

All data will be deposited and accessed on Dryad repository upon acceptance of this paper.
