# Supplementary material for: Functional coordination between leaf traits and biomass allocation and growth of four herbaceous species in a newly established reservoir riparian ecosystem in China
Source: Ecol Evol. 2018 Nov 8;8(23):11372–84. doi: 10.1002/ece3.4494 (PMC6303726; doi:10.1002/ece3.4494)
Supplement: Supplementary file 1 [file ECE3-8-11372-s001.doc]

**Supplementary materials**

**Table S1 Mean effect sizes (*β*) and 95% bootstrap confidence interval (CI) on RGR in four C4 grass species. Bold highlight denotes a statistical significance where 95% CI do not overlap** zero.

| Species | Nutrient | Temperature | N2 | | N5 | | | | N10 | | | |
| --- | --- | --- | --- | --- | --- | --- | --- | --- | --- | --- | --- | --- |
|  |  |  | + 4˚C | | Ambient | | + 4˚C | | Ambient | | + 4˚C | |
|  |  |  | *β* | CI | *β* | CI | *β* | CI | *β* | CI | *β* | CI |
| *Echinochloa crusgalli* | N2 | Ambient | -0.007 | -0.024, 0.009 | 0.003 | -0.013, 0.02 | -0.001 | -0.019, 0.017 | -0.006 | -0.024, 0.01 | -0.01 | -0.027, 0.006 |
|  |  | + 4˚C |  |  | 0.01 | -0.006, 0.028 | 0.006 | -0.011, 0.022 | 0.001 | -0.017, 0.018 | -0.003 | -0.019, 0.014 |
|  | N5 | Ambient |  |  |  |  | -0.004 | -0.021, 0.013 | -0.009 | -0.028, 0.008 | -0.013 | -0.029, 0.003 |
|  |  | + 4˚C |  |  |  |  |  |  | -0.005 | -0.022, 0.012 | -0.009 | -0.026, 0.009 |
|  | N10 | Ambient |  |  |  |  |  |  |  |  | -0.004 | -0.021, 0.014 |
| *Setaria viridis* | N2 | Ambient | **-0.038** | **-0.063, -0.016** | 0 | -0.024, 0.025 | **-0.046** | **-0.07, -0.022** | -0.014 | -0.039, 0.009 | **-0.044** | **-0.069, -0.018** |
|  |  | + 4˚C |  |  | **0.038** | **0.02, 0.056** | -0.007 | -0.026, 0.013 | **0.024** | **0.007, 0.042** | -0.005 | -0.023, 0.014 |
|  | N5 | Ambient |  |  |  |  | **-0.045** | **-0.062, -0.028** | -0.014 | -0.032, 0.004 | **-0.043** | **-0.062, -0.025** |
|  |  | + 4˚C |  |  |  |  |  |  | **0.031** | **0.013, 0.048** | 0.002 | -0.016, 0.02 |
|  | N10 | Ambient |  |  |  |  |  |  |  |  | **-0.03** | **-0.047, -0.012** |
| *Cynodon dactylon* | N2 | Ambient | 0.005 | -0.015, 0.024 | 0.014 | -0.006, 0.035 | **0.022** | **0.002, 0.042** | **0.03** | **0.011, 0.05** | 0.003 | -0.017, 0.025 |
|  |  | + 4˚C |  |  | 0.009 | -0.011, 0.028 | 0.017 | -0.0002, 0.036 | **0.025** | **0.006, 0.044** | -0.002 | -0.022, 0.018 |
|  | N5 | Ambient |  |  |  |  | 0.008 | -0.011, 0.027 | 0.016 | -0.002, 0.036 | -0.011 | -0.032, 0.011 |
|  |  | + 4˚C |  |  |  |  |  |  | 0.008 | -0.012, 0.027 | -0.019 | -0.038, 0.0003 |
|  | N10 | Ambient |  |  |  |  |  |  |  |  | **-0.027** | **-0.048, -0.006** |
| *Hemarthria altissima* | N2 | Ambient | 0.011 | -0.005, 0.027 | 0.015 | -0.001, 0.031 | 0.002 | -0.013, 0.017 | **0.045** | **0.03, 0.061** | 0.004 | -0.012, 0.02 |
|  |  | + 4˚C |  |  | 0.005 | -0.011, 0.021 | -0.008 | -0.025, 0.008 | **0.035** | **0.019, 0.05** | -0.006 | -0.021, 0.01 |
|  | N5 | Ambient |  |  |  |  | -0.013 | -0.028, 0.002 | **0.03** | **0.015, 0.046** | -0.011 | -0.028, 0.004 |
|  |  | + 4˚C |  |  |  |  |  |  | **0.043** | **0.027, 0.059** | 0.002 | -0.013, 0.018 |
|  | N10 | Ambient |  |  |  |  |  |  |  |  | **-0.041** | **-0.057, -0.023** |

**Table S2 Mean difference (*β*) and 95% bootstrap confidence interval (CI) in RGR among four C4 grass species. Bold highlight denotes a statistical significance where 95% CI do not overlap zero.**

| Species | Nutrient | Temperature | *Setaria viridis* | | *Cynodon dactylon* | | *Hemarthria altissima* | |
| --- | --- | --- | --- | --- | --- | --- | --- | --- |
|  |  |  | *β* | CI | *β* | CI | *β* | CI |
| *Echinochloa crusgalli* | 2 | Ambient | -0.015 | -0.040, 0.010 | **-0.044** | **-0.062, -0.026** | **-0.048** | **-0.066, -0.033** |
|  |  | +4˚C | **-0.046** | **-0.063, -0.027** | **-0.032** | **-0.049, -0.015** | **-0.030** | **-0.047, -0.014** |
|  | 5 | Ambient | **-0.018** | **-0.036, -0.0002** | **-0.033** | **-0.051, -0.016** | **-0.036** | **-0.053, -0.0020** |
|  |  | +4˚C | **-0.059** | **-0.077, -0.041** | **-0.021** | **-0.039, -0.002** | **-0.045** | **-0.061, -0.028** |
|  | 10 | Ambient | **-0.023** | **-0.040, -0.006** | -0.008 | -0.027, 0.012 | 0.003 | -0.012, 0.021 |
|  |  | +4˚C | **-0.049** | **-0.066, -0.031** | **-0.031** | **-0.050, -0.012** | **-0.034** | **-0.050, -0.017** |
| *Setaria viridis* | 2 | Ambient |  |  | **-0.029** | **-0.055, -0.003** | **-0.033** | **-0.057, -0.010** |
|  |  | +4˚C |  |  | 0.014 | -0.003, 0.032 | 0.016 | -0.002, 0.033 |
|  | 5 | Ambient |  |  | -0.015 | -0.034, 0.003 | **-0.018** | **-0.035, -0.0001** |
|  |  | +4˚C |  |  | **0.038** | **0.020, 0.057** | 0.014 | -0.027, 0.031 |
|  | 10 | Ambient |  |  | 0.015 | -0.003, 0.034 | **0.026** | **0.011, 0.042** |
|  |  | +4˚C |  |  | 0.018 | -0.003, 0.038 | 0.015 | -0.003, 0.033 |
| *Cynodon dactylon* | 2 | Ambient |  |  |  |  | -0.004 | -0.023, 0.014 |
|  |  | +4˚C |  |  |  |  | 0.002 | -0.016, 0.019 |
|  | 5 | Ambient |  |  |  |  | -0.003 | -0.020, 0.016 |
|  |  | +4˚C |  |  |  |  | **-0.024** | **-0.040, -0.007** |
|  | 10 | Ambient |  |  |  |  | 0.011 | -0.008, 0.029 |
|  |  | +4˚C |  |  |  |  | -0.003 | -0.022, 0.017 |

Figure S1. The multivariate associations of the 9 leaf traitsshown in Table 5 were analyzed with a principal component analysis (PCA) using log10-transformed values of the means of the leaf traits. The bold number in each big circle represents individual species, 1: *Echinochloa crusgalli*; 2: *Setaria viridis*; 3: *Cynodon dactylon*; 4: *Hemarthria altissima*. The three digits besides small circle represent the combination of species + temperature + soil nutrient supply level. For example, 111 represent *E. crusgalli* grown at substrate N=0.4 mg g^-1^, P=0.2 mg g^-1^ at ambient air temperature; 112 represent *E. crusgalli* grown at substrate N=0.5 mg g^-1^, P=0.1 mg g^-1^ at ambient air temperature; 113 represent *E. crusgalli* grown at substrate N=1 mg g^-1^, P=0.1 mg g^-1^ at ambient air temperature; 121 represent *E. crusgalli* grown at substrate N=0.4 mg g^-1^, P=0.2 mg g^-1^ at elevated air temperature; 122 represent *E. crusgalli* grown at substrate N=0.5 mg g^-1^, P=0.1 mg g^-1^ at elevated temperature; 123 represent *E. crusgalli* grown at substrate N=1 mg g^-1^, P=0.1 mg g^-1^ at elevated air temperature.
